# Poverty, Racism, and the Public Health Crisis in America

**DOI:** 10.3389/fpubh.2021.699049

**Published:** 2021-09-06

**Authors:** Bettina M. Beech, Chandra Ford, Roland J. Thorpe, Marino A. Bruce, Keith C. Norris

**Affiliations:** ^1^Department of Health Systems and Population Health Science, University of Houston College of Medicine, Houston, TX, United States; ^2^Department of Community Health Sciences, Center for the Study of Racism, Social Justice and Health at the University of California, Los Angeles, Los Angeles, CA, United States; ^3^Department of Health, Behavior, and Society, Program for Research on Men's Health, Hopkins Center for Health Disparities Solutions, Johns Hopkins Alzheimer's Disease Resource Center for Minority Aging Research, Johns Hopkins Bloomberg School of Public Health, Baltimore, MD, United States; ^4^Program for Research on Faith, Justice, and Health, Department of Behavioral and Social Sciences, University of Houston College of Medicine, Houston, TX, United States; ^5^Department of Medicine, Division of General Internal Medicine and Health Services Research, David Geffen School of Medicine, University of California, Los Angeles, Los Angeles, CA, United States

**Keywords:** racism, structural inequities, poverty, race, social determinants of health

## Abstract

The purpose of this article is to discuss poverty as a multidimensional factor influencing health. We will also explicate how racism contributes to and perpetuates the economic and financial inequality that diminishes prospects for population health improvement among marginalized racial and ethnic groups. Poverty is one of the most significant challenges for our society in this millennium. Over 40% of the world lives in poverty. The U.S. has one of the highest rates of poverty in the developed world, despite its collective wealth, and the burden falls disproportionately on communities of color. A common narrative for the relatively high prevalence of poverty among marginalized minority communities is predicated on racist notions of racial inferiority and frequent denial of the structural forms of racism and classism that have contributed to public health crises in the United States and across the globe. Importantly, poverty is much more than just a low-income household. It reflects economic well-being, the ability to negotiate society relative to education of an individual, socioeconomic or health status, as well as social exclusion based on institutional policies, practices, and behaviors. Until structural racism and economic injustice can be resolved, the use of evidence-based prevention and early intervention initiatives to mitigate untoward effects of socioeconomic deprivation in communities of color such as the use of social media/culturally concordant health education, social support, such as social networks, primary intervention strategies, and more will be critical to address the persistent racial/ethnic disparities in chronic diseases.

*I used to think I was poor, then they told me I wasn't poor, I was needy. Then they told me it was self-defeating to think of myself as needy, I was deprived. Then they told me underprivileged was overused, that I was disadvantaged. I still don't have a dime, but I have a great vocabulary—From a Jules Feiffer cartoon, 1965*.

Poverty is one of the most significant, yet understudied social conditions of the 21st century (1). This social condition can be defined in a number of ways; however, it can be summarized as the lack of resources necessary to meet basic human needs. Prosperity has been a primary focus in recent years with the rise in overall global wealth (2). But, the growth in economic and financial resources has not been equally distributed. The gap in resources between the affluent and the poor has been steadily increasing and global extreme poverty (individual income < United States [U.S.] $1.90/day) increased in 2020, the first time in over two decades to 9.2%, after falling to a low of 8% in 2019 (3). Over 40% of the world lived on less than the U.S. $5.50 a day in 2017 with most of the extreme poverty concentrated in Africa (3). The prevalence of extreme poverty in the U.S. is very low by global standards (3). However, the U.S. has one of the highest rates of poverty in the developed world and the worst index of health and social problems as a function of income inequality (4). For each additional household member, the level increases by $4,480 a year. The level of relative poverty in the U.S. is determined by the federal poverty level (FPL), and for a single-person household, the 2020 poverty level was $12,760 a year, or just under $35 a day. The prevalence of communities being below the FPL varies by race and ethnicity with 24.2% American Indian/Alaskan Native, 21.2% of Black, 17.2% of Hispanic, 9.7% of Asian/Pacific Islander/Native Hawaiian, and 9% of White American families falling below 100% of the FPL (5). Furthermore, the inequities in wealth are even greater than income differences across racial and ethnic groups.

Black families in the U.S. have about one-twentieth the wealth of their White peers on average (6). For every dollar of wealth in White families, the corresponding wealth in Black households is five cents. Wealth inequality is not a function of work ethic or work hour difference between groups. Rather, the widening gap between the affluent and the poor can be linked to unjust policies and practices that favor the wealthy (2, 7–9). The impact of this form of inequality on health has come into sharp focus during the COVID-19 pandemic as the economically disadvantaged were more likely to get infected with SARS CoV-2 and die (10).

For many health providers, the link between poverty and health among health care providers has been primarily grounded in access to health care with several downstream effects of poverty that may include poor nutrition and substandard housing. This understanding is often influenced and perhaps confounded by the correlation between race and poverty, or racism and classism (11). A common narrative for the relatively high prevalence of poverty in marginalized minority communities is predicated on notions about them having poor work ethics and poor innate inabilities to achieve wealth. An over-reliance on the myth of meritocracy and a failure to understand root causes of poverty operating at community and individual levels can exacerbate poor patient-provider relations and perpetuate suboptimal patient outcomes among marginalized minority groups. Racial and economic marginalization has contributed to public and population health crises in the United States (U.S.) and across the globe (12, 13). However, poverty is much more than just a low household income. Poverty has been characterized in the following three ways: (1) economic well-being, commonly linked to income; (2) ability to navigate society as a function of an education or health status of the individual; and/or (3) social exclusion as a result of institutional behaviors, practices, and policies (1, 14). The purpose of this article is to discuss poverty as a multidimensional factor influencing health and explicate how racism contributes to and perpetuates the economic and financial inequality that diminishes prospects for population health improvement among marginalized racial and ethnic groups. We believe this discussion will help to inform a realistic way forward in the pursuit of health equity.

## Poverty and Health Disparities, A Historical Perspective

In the mid-1800's, Dr. James McCune Smith was the leading voice in the medical profession to argue that the health of the person was not primarily a consequence of their innate constitution, but instead reflected their intrinsic membership in groups created by a race structured society (15–17). This articulation of health disparities being linked to the racial caste system of America and inequitable social conditions is one of the earliest written descriptions of racism as the cause of health inequities and ultimately health disparities by a member of the American healthcare community. His arguments were scientifically validated when Dr. William Edward Burghardt Dubois reported his findings in 1899 from the first sociological study of Blacks in America, The Philadelphia Negro, demonstrating that racial differences in mortality in Philadelphia were explained by social factors (e.g., economic, sanitary, and education) and not innate racial traits or tendencies (18). Dr. Dubois documented how white supremacy policies, actions, and beliefs leading to discrimination, oppression, and more contributed to structural poverty and increased levels of despair, disease, and death (19). Thus, Drs. James and Dubois are considered by many to be the true pioneers who laid the foundation for future work clarifying racism rather than race as the cause of health disparities (20, 21).

Explicit notions of Black biological, intellectual, and moral inferiority often categorized as scientific racism have gradually moved from the mainstream to the margins over the last century as social movements advocated for the full citizenship of Black Americans. Despite the passing of civil rights legislation in the U.S. prohibiting discrimination in public arenas and civic engagement (e.g., Civil Rights Bill of 1866, Civil Rights Act of 1964, Voting Rights Act of 1965, and Civil Rights Act of 1968), structural racism, discrimination, and other harmful forms of bias continue to persist today (22–24). Many factors, such as explicit and implicit provider biases, medical and institutional mistrust (due to historic and contemporary mistreatment), and low self-esteem and stereotype threat, from internalized racism continue to impact our nation and further contribute to the genesis and perpetuation of health disparities (25). This was reified in the 1985 Report of the Secretary of the U.S. Department of Health and Human Services (U.S. DHHS) Task Force on Black and Minority Health, known as the Heckler Report, the first government-sanctioned assessment of racial health disparities (26), followed nearly two decades thereafter by the Institute of Medicine (IOM) Report on Unequal Treatment (25). The Heckler Report noted mortality inequity was linked to six leading causes of preventable excess deaths for the Black compared to the White population (cancer, cardiovascular disease, diabetes, infant mortality, chemical dependency, and homicide/unintentional injury) (26). The IOM Report focused on health care disparities and highlighted the role interpersonal racism can have on health outcomes for members of minoritized groups (25). These reports and others (9, 27–35) have led to a more robust focus on population health over the last few decades that has included a renewed interest in the impact of racism and social factors, such as poverty on clinical outcomes (1, 33).

## Poverty and the MYTH of Meritocracy

The race is an antecedent and major determinant of socioeconomic status (SES) in the U.S.; therefore, it is not surprising that the successful implementation of discriminatory race-based policies premised on racial inferiority would produce racial disparities in SES. The term structural racism is used to capture the ways in which inequities are perpetuated through the racialized differential access to resources, opportunities, and services that are codified in laws, policies, practices, and societal norms (23, 32, 33, 36–40). This system harms marginalized populations at the expense of affording greater resources, opportunities, and other privileges to the dominant White society (23, 32, 33, 36–40). Importantly, a single identifiable perpetrator is not visible making its denial easy and its identification and dissolution challenging (41).

However, the role of structural racism in creating and sustaining poverty is rarely discussed in scholarly and public circles despite the publishing of seminal works, such as *Caste, Class and Race, Black Metropolis*, and *An American Dilemma* during the mid-20th century (42–44). These groundbreaking books laid the foundation for several sociological studies documenting key structurally racist policies and practices (i.e., residential segregation) that created communities comprised of racial and ethnic minorities that are beset with poverty and related factors, including high unemployment, poor schools, substandard housing, and limited social mobility (45–47). Most White Americans were not exposed to this scholarship nor the overwhelming financial and economic disadvantages faced by African Americans and other marginalized groups. As such, public discourse has been largely shaped by a narrative of meritocracy which is laced with ideals of opportunity without any consideration of the realities of racism and race-based inequities in structures and systems that have locked individuals, families, and communities into poverty-stricken lives for generations. Pervasive public policies spanning from slavery to voter suppression have and continue to severely limit opportunities for social mobility among marginalized groups, thereby perpetuating and hardening vast inequities in power, status, and resources that define our racial caste system and structure (9, 34, 48–50).

The narrative of meritocracy has also been extended to immigrants, but it is framed through a narrative of European immigrants who work hard and become successful. However, immigrants from Mexico, Central and South America, in particular as well as many refugees from poor Asian and African countries are also exposed to laws and policies that create and perpetuate a life confronting persistent inequality and perceptions of inferiority.

These practices of race-based, community-level disinvestment in each of the domains of the social determinants coupled with a lack of a national health program condemn oppressed populations such as Black and Hispanic Americans, American Indians, and disproportionately non-English speaking immigrants and refugees to remain in poverty and suffer from suboptimal health. Thus, poverty represents a critical public health condition that is both determined by and perpetuated by structural racism.

## Conceptual Framework of Poverty and Health

**Figure 1:**
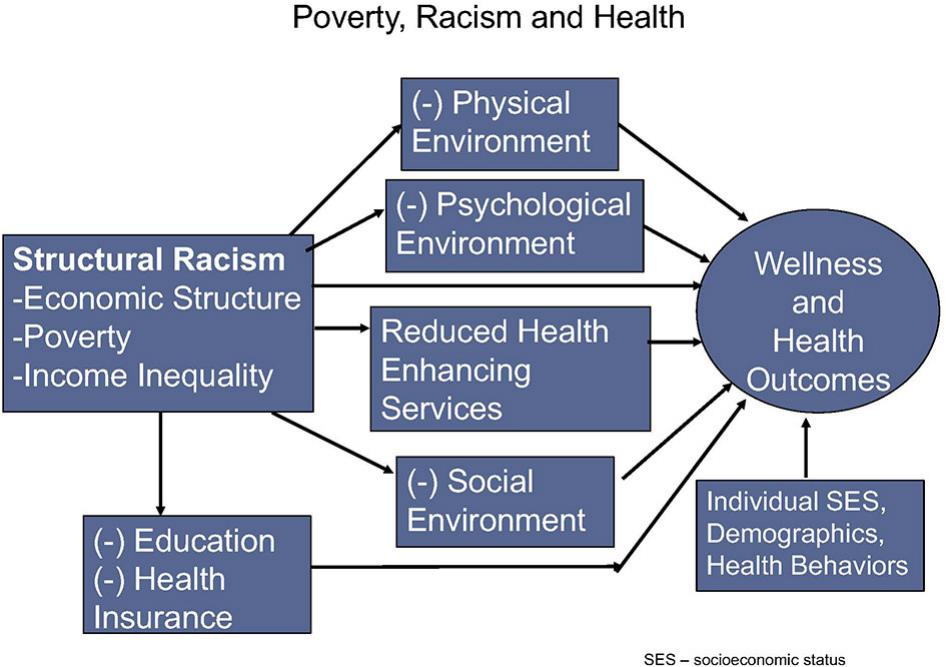
A conceptual framework emphasizing the key pathways through which poverty and structural racism may influence wellness and health outcomes [adapted from Wen et al. (55)].

Socioeconomically disadvantaged populations across the globe bear a disproportionate burden of chronic diseases and are least likely to receive evidence-based care leading to optimal clinical outcomes (51, 52). A basic understanding of the vulnerabilities of the marginalized and oppressed populations will facilitate the adaptation and adoption of the necessary policies to support disease treatment and prevention guidelines (52). The WHO has identified three key tenets to improving health at a global level that each reinforces the impact of socioeconomic factors: (1) improve the conditions of daily life; (2) tackle the inequitable distribution of power, money, and resources, the structural drivers of those conditions of daily life, globally, nationally, and locally; and (3) develop a workforce trained in the social determinants of health and raise public awareness about social needs and the social determinants of health (53). Social factors and health behaviors have contributed substantially to the growing non-communicable disease epidemics (e.g., obesity, diabetes, hypertension, and mental health disorders). A deeper understanding and integration of social and behavioral sciences is needed to equip medical and public health communities to address the challenge of providing quality care in the setting of contrasting financial and public health policies to control costs (54). A conceptual framework emphasizing the key pathways through which poverty and structural racism may influence wellness and health outcomes is shown in Figure 1.

## Perniciousness of Persistent Poverty

The Social Determinants of Health (SDoH) are macro-level factors that shape the economic, physical, psychological, and social environments in which people live (56). They are often viewed as having the capacity to enhance or diminish the resources available to individuals to promote health, including but not limited to the food supply, housing, economic and social relationships, transportation, employment, criminal justice, education, and health care, whose distribution across populations effectively determines length and quality of life and the programs and policies that direct them (23). The World Health Organization Commission on Social Determinants of Health has found that the poor health status of low resource persons, communities, and nations is directly related to the unequal distribution of power, income, goods, and services (53). Social structures and institutions with unequal and unfair social policies, economic arrangements, and practices have contributed to much of the health inequity present in the world. A brief overview of select medical conditions follows.

Communicable diseases: Poverty can contribute to many communicable diseases including many acute and chronic infectious diseases. Poverty and the associated disadvantage of personal and social resources often lead to unsafe habitation and lack of cleanliness, unhealthy diets, and malnutrition (including maternal-fetal), poor water quality, increased exposure to infectious diseases, environmental pollution and toxins, and more (57). The rates of infectious disease morbidity and mortality in low resource households, communities, and nations over decades bear witness to the considerable impact of economic inequality on health (53).

Maternal and child health: Poverty has been strongly linked with poor reproductive outcomes, both independently and in combination with exposure to discrimination (58–61). Maternal and child health among low-income and racial/ethnic minority groups are particularly susceptible to psychological stress, nutrition, substance use, and more (58, 62, 63).

Incarcerated youth: Globally and in the US, incarceration rates are higher among poor and marginalized groups (64). For children within the criminal justice system or otherwise deprived of liberty are at particularly high risk of violence, rape and sexual assault, sexually transmitted diseases, substance use disorders, mental illnesses, and physical disorders, many of which will continue throughout the life course (65, 66). Furthermore, adult incarceration can create health deficits in familial youth (64).

Chronic non-communicable diseases: Similar patterns of disparities negatively are observed in the incidence and prevalence of chronic diseases, such as cardiovascular disease, diabetes, kidney disease, and others (67–70). Poverty can also have indirect implications for health (5, 71). Race-based economic disadvantages can influence other social determinants as the intersection with poverty can further limit housing, educational, and employment opportunities, and these have also been linked to worse health outcomes (58, 72–74). Poverty can also influence individual perceptions and behaviors (75). Relative and absolute economic deprivation can shape expectations and perceived life chances in a manner that individuals focus on surviving rather than thriving.

Mental health: In addition to the more traditional mental health conditions that may limit daily functioning, the additional chronic stress associated with navigating basic needs in a state of poverty can impair cognitive processing and the ability to remember and to perform implementation tasks (76), along with mistrust which may impact the ability of the individual to follow up on medical appointments, provider recommendations and more to conspire to limit health outcomes (77).

In summary, the impact of poverty on both the physical and psychological aspects of a person can play an important role in the many dimensions associated with the development and progression of diseases. The socioeconomic status of the individual may considerably impact the perception of the individual of many life issues, such as food, education, language, and time (75). While these concepts may be apparent and easily recognizable in other social disciplines, their presence and implications may be lost or concealed to many health care providers. Therefore, an understanding of how poverty may influence worldviews is critical for health professionals to truly understand the diverse group of patients they care for and how to better connect with those in an impoverished situation to optimize the effectiveness of traditional and alternative health strategies and recommendations. Table 1 highlights the influence of socioeconomic class including income on the context of patient-specific needs, values, and preferences, as well as considerations as to how racism may be operating in that setting.

**Table 1:** Socioeconomic class and values of key determinants of health [adapted from Payne and Blair (75)].

	**Poverty**	**Middle class**	**Wealth**	**Role of racism**
Food	Key question: Did you have enough? Quantity important.	Key question: Did you like it? Quality important.	Key question: Was it presented well? Presentation important.	Key question: Why do poor and mostly non-White people like to eat that way?
Education	Valued and revered as abstract but not as reality.	Crucial for climbing the success ladder and making money.	Necessary tradition for making and maintaining connections.	Maintain substandard resources for schooling in Black and other minoritized school districts thereby perpetuating inequitable higher education and employment opportunities
Destiny	Believes in fate. Cannot do much to mitigate chance.	Believes in choice. Can change future with good choices now.	Noblesse oblige.	Reinforce destiny to a lower caste as fate, grounded in innate group differences
Language	Casual register. Language is about survival.	Formal register. Language is about negotiation.	Formal register. Language is about networking.	Promote narratives that focus on survival for poor and mostly non-White people and reinforce the situation is innate and not due to the racialized caste structures of society
Family structure	Tend to be matriarchal.	Tends to be patriarchal.	Depends on who has money.	Promote laws and policies that require a fractured family setting to be eligible for safety net resources and perpetuate incarceration programs to target and generate and maintain matriarchal social structures in poor and mostly non-White communities
World view	Sees the world in terms of local settings.	Sees the world in terms of national settings.	Sees the world in terms of international view.	Ensure as many as possible poor and mostly non-White people see the plight in their local setting as fixed and due to their innate inferiority
Time	Present most important. Decisions made for the moment based on feelings or survival.	Future most important. Decisions made against future ramifications.	Traditions and history most important. Decisions made partially on the basis of tradition and decorum	Promote narratives that equity and justice to always come a little later. To be patient and that now is never the right time

### Poverty, Refugee, and Migration

As a large nation founded by immigrants, the United States inevitably and receives a large number of refugees, documented, and undocumented immigrants seeking a better life. The national narrative is that immigrants will find employment, gain some measure of socioeconomic equity and become eligible for health insurance. Unfortunately, this ideal only holds true for a subset of preferred immigrants largely from wealthy European countries. Individuals from formerly colonized nations in Central or South America, Asia, or Africa who come to the United States are often beset with persistent marginalization, poverty, and poor health (78, 79). Furthermore, the likelihood that groups will be placed in such a situation is grounded in racial and ethnic discrimination as well as religious discrimination (11). Many immigrants with limited resources experience a combination of stressors, including discrimination, isolation, uncertainty, and mental health disorders from posttraumatic stress symptoms, depression, anxiety alcohol, and substance use to posttraumatic stress symptoms (80, 81). In addition to researchers, providers have acknowledged the importance of poverty, discrimination, and other structural barriers on the lived experiences of immigrant clients and how it may impact their health (80).

## What Might be the Way Forward?

An aphorism commonly attributed to the former Center for Medicare and Medicaid Services (CMS) director Don Berwick is “Every system is perfectly designed to achieve the results it gets.” Our society has been outstanding in perpetuating the conditions that lead to and maintain poverty for a disproportionately high percentage of people of color. Unlike many narratives about poverty and the innate values of people of color, no one wakes up wanting to be poor or sick. Similar to most other major institutions, the health profession has chosen to work around the margins of poverty and to study and practice what is the best way to treat patients with limited resources, limited social support, and multiple exposures that develop or worsen the disease. While the stature of the health profession has given it an immense level of privilege and power that could be used to achieve different results in a nation with immense wealth, we have chosen as a collective not to address the root causes because it would conflict with the white supremacy ideology of a caste-based society. Continuing the same approach to medical education in the setting of our rapidly increasing wealth gap will lead to training physicians and other healthcare providers on how to most effectively care for fewer and fewer people. Creating a new generation of healthcare providers dedicated to mitigating the many social factors that conspire to perpetuate health disparities is one important step toward how the profession can rebuild patient trust and ultimately improve patient outcomes.

The solutions must involve stakeholders from across diverse sectors (82). The medical community and related stakeholders should adopt a strategic approach to address the financial and related public policy issues that will enable the delivery of appropriate clinical care to marginalized patient populations including low those with low SES, minoritized communities, and non-European immigrants and refugees (40, 48, 54, 83). The Affordable Care Act (ACA) was one such policy that dramatically increased the insurance coverage eligibility for a large number of low-income young Americans (84), with important consequences for mitigating health disparities as well as possibly reducing bankruptcy related to health care costs (85), although other data suggest that there has been no impact on bankruptcy (86). Barcellos et al. (87) reported persons with a lower income (100–250% FPL) were 31% less likely to score above the median on ACA knowledge and 54% less likely to score above the median on health insurance knowledge vs. persons with higher income levels (>400% FPL). These findings highlight the need to not only implement health policies to increase access to care for lower-income individuals but also the need to ensure such policies and associated programs are reaching those in need. The ACA may set the stage for not only more available care but also more structured medical care systems which can help improve health outcomes (88). However, improved outreach and education of the potential benefits of and access to the ACA in lower-income communities and support to ensure people are enrolled is still required (87).

A major challenge for the broader medical community is to reconceptualize how it might improve each domain that impacts health outcomes, beyond those limited to a procedure or prescription. Increasing the awareness of environmental and social factors that contribute to health disparities must be followed by actions, such as cost-effective policies, to improve disease prevention and care in impoverished communities, especially in the setting of increasing inequities in wealth and many of the other SDoH (88–92). Healthcare providers can directly address many of the factors crucial for closing the health disparities gap by recognizing and trying to mitigate the race-based implicit biases many physicians carry (93), as well as leveraging their privilege to address the elements of institutionalized racism entrenched within the fabric of our society, starting with social injustice and human indifference (91, 94). Examples of evidence-based initiatives to mitigate untoward effects of socioeconomic deprivation include the use of videos and/or novellas (95, 96), the use of social support, such as social networks (97), and primary intervention strategies including the use of mobile clinics, lay health workers, and patient navigators to address chronic diseases (98–101). Finally, the healthcare sector should not miss the opportunity to learn important lessons as it strives to advance the necessary policies to improve social welfare and health outcomes, as the existence of health inequities provides unique, unrecognized opportunities for understanding biological, environmental, sociocultural, and healthcare system factors that can improve clinical outcomes (88–92).

“*Overcoming poverty is not a gesture of charity. It is an act of justice. It is the protection of a fundamental human right, the right to dignity and a decent life”—Nelson Mandela former President of South Africa*.

## Data Availability Statement

The original contributions presented in the study are included in the article/supplementary material, further inquiries can be directed to the corresponding author.

## Author Contributions

KN wrote the first draft of the manuscript. BB, MB, CF, and RT wrote sections of the manuscript. All author contributed to conception and design of the study, contributed to manuscript revision, read, and approved the submitted version.

## Conflict of Interest

The authors declare that the research was conducted in the absence of any commercial or financial relationships that could be construed as a potential conflict of interest.

## Publisher's Note

All claims expressed in this article are solely those of the authors and do not necessarily represent those of their affiliated organizations, or those of the publisher, the editors and the reviewers. Any product that may be evaluated in this article, or claim that may be made by its manufacturer, is not guaranteed or endorsed by the publisher.
